# Supplementary material for: Single Cell Transcriptomic and Chromatin Profiles Suggest Layer Vb Is the Only Layer With Shared Excitatory Cell Types in the Medial and Lateral Entorhinal Cortex
Source: Front Neural Circuits. 2022 Jan 26;15:806154. doi: 10.3389/fncir.2021.806154 (PMC8826650; doi:10.3389/fncir.2021.806154)
Supplement: Supplementary file 14 [file Data_Sheet_1.ZIP › LEC_Sup_vs_MEC_Sup_Motifs/knownResults.html]

LEC\_Sup\_vs\_MEC\_Sup\_Motifs/ - Homer Known Motif Enrichment Results


# Homer Known Motif Enrichment Results (LEC\_Sup\_vs\_MEC\_Sup\_Motifs/)

Homer *de novo* Motif Results  
Gene Ontology Enrichment Results  
Known Motif Enrichment Results (txt file)  
Total Target Sequences = 13336, Total Background Sequences = 19927

|  |  |  |  |  |  |  |  |  |  |  |  |
| --- | --- | --- | --- | --- | --- | --- | --- | --- | --- | --- | --- |
| Rank | Motif | Name | P-value | log P-pvalue | q-value (Benjamini) | # Target Sequences with Motif | % of Targets Sequences with Motif | # Background Sequences with Motif | % of Background Sequences with Motif | Motif File | SVG |
| 1 | C A T G G T A C G A C T G C T A C G T A C G T A C G T A G C T A G A C T C T G A T C A G G T A C | Mef2c(MADS)/GM12878-Mef2c-ChIP-Seq(GSE32465)/Homer | 1e-97 | -2.241e+02 | 0.0000 | 4098.0 | 30.73% | 4549.3 | 22.83% | motif file (matrix) | svg |
| 2 | C A T G A G T C A G C T C G T A C G A T C G A T G C A T G C A T C G A T C T G A C A T G T G A C | Mef2d(MADS)/Retina-Mef2d-ChIP-Seq(GSE61391)/Homer | 1e-96 | -2.227e+02 | 0.0000 | 2109.0 | 15.82% | 1988.2 | 9.98% | motif file (matrix) | svg |
| 3 | C A G T T C A G G A T C A C T G A C G T C T A G A C T G A C T G G A C T C T A G | Egr1(Zf)/K562-Egr1-ChIP-Seq(GSE32465)/Homer | 1e-93 | -2.153e+02 | 0.0000 | 2544.0 | 19.08% | 2544.9 | 12.77% | motif file (matrix) | svg |
| 4 | G T A C G A T C C A G T A G T C A G T C A G T C T G C A G A T C C T G A A T G C G T C A A C G T | WT1(Zf)/Kidney-WT1-ChIP-Seq(GSE90016)/Homer | 1e-84 | -1.946e+02 | 0.0000 | 2341.0 | 17.56% | 2344.7 | 11.77% | motif file (matrix) | svg |
| 5 | C A T G A G T C G A C T C G T A C G A T G C A T G A C T G C A T C G A T C T A G C A T G T G A C | Mef2b(MADS)/HEK293-Mef2b.V5-ChIP-Seq(GSE67450)/Homer | 1e-79 | -1.828e+02 | 0.0000 | 5937.0 | 44.53% | 7279.7 | 36.54% | motif file (matrix) | svg |
| 6 | T C A G C T A G A T G C G A C T T G C A C G T A A G C T A C G T C T G A T A C G | En1(Homeobox)/SUM149-EN1-ChIP-Seq(GSE120957)/Homer | 1e-68 | -1.578e+02 | 0.0000 | 8091.0 | 60.68% | 10586.9 | 53.13% | motif file (matrix) | svg |
| 7 | A T C G A T C G A T G C G A C T T C G A C G T A G C A T A G C T C T G A T A C G | DLX2(Homeobox)/BasalGanglia-Dlx2-ChIP-seq(GSE124936)/Homer | 1e-63 | -1.458e+02 | 0.0000 | 7400.0 | 55.50% | 9604.2 | 48.20% | motif file (matrix) | svg |
| 8 | C T A G T C A G C A G T T C A G A C T G A C T G G A T C C T A G A C T G C T A G T C A G A T G C | KLF14(Zf)/HEK293-KLF14.GFP-ChIP-Seq(GSE58341)/Homer | 1e-55 | -1.286e+02 | 0.0000 | 4027.0 | 30.20% | 4819.7 | 24.19% | motif file (matrix) | svg |
| 9 | C G T A C G T A A G C T G A C T T G C A G T C A A C G T A G C T C T G A T C A G | Lhx3(Homeobox)/Neuron-Lhx3-ChIP-Seq(GSE31456)/Homer | 1e-54 | -1.263e+02 | 0.0000 | 7594.0 | 56.95% | 9998.6 | 50.18% | motif file (matrix) | svg |
| 10 | A T C G A T G C A G T C T A G C G A C T T C G A G C T A G C A T A G C T C T G A | DLX1(Homeobox)/BasalGanglia-Dlx1-ChIP-seq(GSE124936)/Homer | 1e-54 | -1.244e+02 | 0.0000 | 6730.0 | 50.47% | 8720.7 | 43.77% | motif file (matrix) | svg |
| 11 | A T G C T A C G A G T C G A C T T G C A C T G A G A C T A C G T C T G A T C A G | LHX9(Homeobox)/Hct116-LHX9.V5-ChIP-Seq(GSE116822)/Homer | 1e-53 | -1.235e+02 | 0.0000 | 6836.0 | 51.27% | 8882.5 | 44.58% | motif file (matrix) | svg |
| 12 | A C T G C A G T A C G T C G T A C G T A A C G T A C T G C T G A | Nkx6.1(Homeobox)/Islet-Nkx6.1-ChIP-Seq(GSE40975)/Homer | 1e-50 | -1.157e+02 | 0.0000 | 9731.0 | 72.98% | 13346.5 | 66.98% | motif file (matrix) | svg |
| 13 | A G C T G T C A C G T A A C G T A C G T C T G A T C A G A T G C | Lhx2(Homeobox)/HFSC-Lhx2-ChIP-Seq(GSE48068)/Homer | 1e-46 | -1.077e+02 | 0.0000 | 5883.0 | 44.12% | 7571.8 | 38.00% | motif file (matrix) | svg |
| 14 | G T A C G A C T C G T A C T G A T C G A C G T A G C T A C A G T C T G A T A C G | Mef2a(MADS)/HL1-Mef2a.biotin-ChIP-Seq(GSE21529)/Homer | 1e-45 | -1.058e+02 | 0.0000 | 3312.0 | 24.84% | 3937.1 | 19.76% | motif file (matrix) | svg |
| 15 | C G T A A C T G C G T A A C G T C G T A C T G A T C A G T G C A A G C T T G A C | TRPS1(Zf)/MCF7-TRPS1-ChIP-Seq(GSE107013)/Homer | 1e-43 | -1.012e+02 | 0.0000 | 7465.0 | 55.98% | 9950.7 | 49.94% | motif file (matrix) | svg |
| 16 | C G T A C A T G A G T C G A C T T G C A C G T A A C G T A C G T C T G A T C A G | Lhx1(Homeobox)/EmbryoCarcinoma-Lhx1-ChIP-Seq(GSE70957)/Homer | 1e-42 | -9.781e+01 | 0.0000 | 6080.0 | 45.60% | 7917.2 | 39.73% | motif file (matrix) | svg |
| 17 | A C T G C T A G T C G A C G A T C A T G G C T A A T C G C G A T G T A C G C T A A G C T G T A C | Fra1(bZIP)/BT549-Fra1-ChIP-Seq(GSE46166)/Homer | 1e-42 | -9.700e+01 | 0.0000 | 3420.0 | 25.65% | 4128.3 | 20.72% | motif file (matrix) | svg |
| 18 | G C T A A T C G G C T A G A C T G C T A T C G A T A G C T C G A | GATA3(Zf)/iTreg-Gata3-ChIP-Seq(GSE20898)/Homer | 1e-41 | -9.559e+01 | 0.0000 | 5751.0 | 43.13% | 7450.4 | 37.39% | motif file (matrix) | svg |
| 19 | A C T G C A T G T C G A A C G T A C T G C G T A A T C G A C G T G T A C C G T A G A C T A G T C | Fos(bZIP)/TSC-Fos-ChIP-Seq(GSE110950)/Homer | 1e-40 | -9.312e+01 | 0.0000 | 3571.0 | 26.78% | 4357.7 | 21.87% | motif file (matrix) | svg |
| 20 | C A G T T G C A A C G T A C T G C G T A A T C G C G A T T G A C C G T A A C G T | BATF(bZIP)/Th17-BATF-ChIP-Seq(GSE39756)/Homer | 1e-39 | -9.167e+01 | 0.0000 | 3767.0 | 28.25% | 4638.3 | 23.28% | motif file (matrix) | svg |
| 21 | A T G C T A C G A G C T T G C A C G T A C G A T A C G T C T G A | DLX5(Homeobox)/BasalGanglia-Dlx5-ChIP-seq(GSE124936)/Homer | 1e-39 | -9.053e+01 | 0.0000 | 4770.0 | 35.77% | 6064.7 | 30.44% | motif file (matrix) | svg |
| 22 | C T A G T C G A G C A T C A T G G C T A T A G C C G A T G T A C C T G A A G C T | JunB(bZIP)/DendriticCells-Junb-ChIP-Seq(GSE36099)/Homer | 1e-39 | -9.037e+01 | 0.0000 | 3265.0 | 24.49% | 3947.8 | 19.81% | motif file (matrix) | svg |
| 23 | C T A G T C G A A C G T A C T G C G T A A T G C A C G T G T A C C G T A A G C T G A T C G T A C | Atf3(bZIP)/GBM-ATF3-ChIP-Seq(GSE33912)/Homer | 1e-37 | -8.694e+01 | 0.0000 | 3773.0 | 28.30% | 4672.5 | 23.45% | motif file (matrix) | svg |
| 24 | T A C G A T G C G C T A A C T G C G T A A C G T C G T A C T G A T A C G T C G A | Gata4(Zf)/Heart-Gata4-ChIP-Seq(GSE35151)/Homer | 1e-37 | -8.562e+01 | 0.0000 | 3908.0 | 29.31% | 4869.2 | 24.44% | motif file (matrix) | svg |
| 25 | G C A T G C A T C T G A A C G T C T G A A C G T C G T A C G T A C G T A A G T C G T C A G T C A | Foxf1(Forkhead)/Lung-Foxf1-ChIP-Seq(GSE77951)/Homer | 1e-36 | -8.289e+01 | 0.0000 | 5411.0 | 40.58% | 7035.6 | 35.31% | motif file (matrix) | svg |
| 26 | A G C T A T G C G A C T G C A T C G T A A G C T G T A C C G A T A T C G A G T C | Gata6(Zf)/HUG1N-GATA6-ChIP-Seq(GSE51936)/Homer | 1e-34 | -7.905e+01 | 0.0000 | 3648.0 | 27.36% | 4541.3 | 22.79% | motif file (matrix) | svg |
| 27 | T C G A C A G T A C T G A G C T C G T A C G T A A C G T A C G T C T G A T A G C | Dlx3(Homeobox)/Kerainocytes-Dlx3-ChIP-Seq(GSE89884)/Homer | 1e-34 | -7.830e+01 | 0.0000 | 4345.0 | 32.59% | 5531.6 | 27.76% | motif file (matrix) | svg |
| 28 | A C G T T G C A A G C T G A T C C T A G C T G A A G C T G T C A T C G A C G T A | CUX1(Homeobox)/K562-CUX1-ChIP-Seq(GSE92882)/Homer | 1e-33 | -7.766e+01 | 0.0000 | 2865.0 | 21.49% | 3461.6 | 17.37% | motif file (matrix) | svg |
| 29 | T C G A A C G T C A T G G C T A T A G C C G A T G T A C G C T A A C G T A T G C | AP-1(bZIP)/ThioMac-PU.1-ChIP-Seq(GSE21512)/Homer | 1e-33 | -7.671e+01 | 0.0000 | 3962.0 | 29.71% | 4997.1 | 25.08% | motif file (matrix) | svg |
| 30 | A C G T T A C G G A T C A C T G A C G T C T A G A C T G A C T G G A T C C T A G C A T G C T A G | Egr2(Zf)/Thymocytes-Egr2-ChIP-Seq(GSE34254)/Homer | 1e-33 | -7.653e+01 | 0.0000 | 645.0 | 4.84% | 580.6 | 2.91% | motif file (matrix) | svg |
| 31 | C A T G C T A G T C G A A C G T A C T G C G T A T A G C C G A T T G A C C G T A A G C T G A T C | Fra2(bZIP)/Striatum-Fra2-ChIP-Seq(GSE43429)/Homer | 1e-32 | -7.372e+01 | 0.0000 | 2879.0 | 21.59% | 3501.8 | 17.57% | motif file (matrix) | svg |
| 32 | A G C T A G T C A T G C A G C T A C G T C G T A A C G T A G T C C G A T A T G C | Gata2(Zf)/K562-GATA2-ChIP-Seq(GSE18829)/Homer | 1e-29 | -6.777e+01 | 0.0000 | 2516.0 | 18.87% | 3034.1 | 15.23% | motif file (matrix) | svg |
| 33 | C T A G T C G A C G A T A C T G C G T A T A C G A G C T T G A C G C T A A C G T G A T C T A G C | Fosl2(bZIP)/3T3L1-Fosl2-ChIP-Seq(GSE56872)/Homer | 1e-28 | -6.602e+01 | 0.0000 | 2070.0 | 15.52% | 2437.2 | 12.23% | motif file (matrix) | svg |
| 34 | C T A G A C T G C T A G T C A G T C A G T A C G C T A G A C T G | Maz(Zf)/HepG2-Maz-ChIP-Seq(GSE31477)/Homer | 1e-27 | -6.398e+01 | 0.0000 | 2759.0 | 20.69% | 3390.0 | 17.01% | motif file (matrix) | svg |
| 35 | C G T A G C T A C G A T C T A G A C G T G T C A C G T A C G T A A G T C C G T A T G C A T A C G | FoxL2(Forkhead)/Ovary-FoxL2-ChIP-Seq(GSE60858)/Homer | 1e-26 | -6.192e+01 | 0.0000 | 4864.0 | 36.48% | 6386.4 | 32.05% | motif file (matrix) | svg |
| 36 | C T A G T C G A A C G T A C T G C G T A T A G C C G A T G T A C C G T A A G C T G A T C G T A C | Jun-AP1(bZIP)/K562-cJun-ChIP-Seq(GSE31477)/Homer | 1e-26 | -6.117e+01 | 0.0000 | 1605.0 | 12.04% | 1838.7 | 9.23% | motif file (matrix) | svg |
| 37 | T A G C G C T A A C T G C G T A A C G T C G T A C G T A T A C G T C A G T C G A | Gata1(Zf)/K562-GATA1-ChIP-Seq(GSE18829)/Homer | 1e-26 | -6.048e+01 | 0.0000 | 2150.0 | 16.12% | 2573.7 | 12.92% | motif file (matrix) | svg |
| 38 | C G A T C T A G A C G T G T C A C G T A C G T A A G T C C G T A | Foxo3(Forkhead)/U2OS-Foxo3-ChIP-Seq(E-MTAB-2701)/Homer | 1e-25 | -5.846e+01 | 0.0000 | 4097.0 | 30.73% | 5308.3 | 26.64% | motif file (matrix) | svg |
| 39 | C T G A T C A G C A G T C T A G A C T G C T A G G A T C A T C G A C T G C T G A T C A G G A T C | Sp5(Zf)/mES-Sp5.Flag-ChIP-Seq(GSE72989)/Homer | 1e-25 | -5.790e+01 | 0.0000 | 2129.0 | 15.97% | 2558.5 | 12.84% | motif file (matrix) | svg |
| 40 | A G T C C G A T C T G A C G T A A C G T C A G T T C A G T G A C | Isl1(Homeobox)/Neuron-Isl1-ChIP-Seq(GSE31456)/Homer | 1e-25 | -5.762e+01 | 0.0000 | 7442.0 | 55.81% | 10222.2 | 51.30% | motif file (matrix) | svg |
| 41 | C T G A C T G A C A T G A T C G A G C T A T C G G A C T C A T G C T G A G T C A | Tbr1(T-box)/Cortex-Tbr1-ChIP-Seq(GSE71384)/Homer | 1e-24 | -5.692e+01 | 0.0000 | 6000.0 | 45.00% | 8083.4 | 40.57% | motif file (matrix) | svg |
| 42 | A G C T A G T C A G T C A C G T C T A G A C G T A C G T A C G T C G T A A G T C G A T C C G T A | FOXP1(Forkhead)/H9-FOXP1-ChIP-Seq(GSE31006)/Homer | 1e-21 | -4.912e+01 | 0.0000 | 2505.0 | 18.79% | 3126.5 | 15.69% | motif file (matrix) | svg |
| 43 | A C T G T C A G A G C T G A C T C A T G A G T C A G T C G C T A C G A T C T A G T C A G G T A C C T G A T C G A | Rfx1(HTH)/NPC-H3K4me1-ChIP-Seq(GSE16256)/Homer | 1e-20 | -4.813e+01 | 0.0000 | 1193.0 | 8.95% | 1352.4 | 6.79% | motif file (matrix) | svg |
| 44 | A G C T T G A C C G A T C G A T C T A G A C G T C A G T C A G T G C T A A G T C | FOXK1(Forkhead)/HEK293-FOXK1-ChIP-Seq(GSE51673)/Homer | 1e-20 | -4.777e+01 | 0.0000 | 5250.0 | 39.37% | 7059.7 | 35.43% | motif file (matrix) | svg |
| 45 | G C T A T C G A C G T A C T A G A G C T G T C A G T C A C G T A A G T C C G T A | FOXA1(Forkhead)/MCF7-FOXA1-ChIP-Seq(GSE26831)/Homer | 1e-19 | -4.501e+01 | 0.0000 | 5263.0 | 39.47% | 7102.3 | 35.65% | motif file (matrix) | svg |
| 46 | T C A G T G A C G T A C T G C A G T A C C T A G G T A C A T G C A G T C G T C A A G T C G A C T | Klf9(Zf)/GBM-Klf9-ChIP-Seq(GSE62211)/Homer | 1e-19 | -4.500e+01 | 0.0000 | 896.0 | 6.72% | 979.2 | 4.91% | motif file (matrix) | svg |
| 47 | G A C T G T A C T G C A A C G T G A T C G C T A T C G A A C G T A G T C C G T A | Pdx1(Homeobox)/Islet-Pdx1-ChIP-Seq(SRA008281)/Homer | 1e-19 | -4.452e+01 | 0.0000 | 4518.0 | 33.88% | 6022.8 | 30.23% | motif file (matrix) | svg |
| 48 | A T G C A G T C G T A C A G C T T C G A C T A G G A T C C T G A G T C A A G T C G C T A T C A G | Rfx5(HTH)/GM12878-Rfx5-ChIP-Seq(GSE31477)/Homer | 1e-18 | -4.290e+01 | 0.0000 | 1738.0 | 13.03% | 2108.6 | 10.58% | motif file (matrix) | svg |
| 49 | G C T A T C G A C G T A C T A G A G C T G T C A G T C A C G T A A G T C C G T A | FOXA1(Forkhead)/LNCAP-FOXA1-ChIP-Seq(GSE27824)/Homer | 1e-18 | -4.264e+01 | 0.0000 | 6063.0 | 45.47% | 8300.5 | 41.66% | motif file (matrix) | svg |
| 50 | C T A G A G C T G A C T C A T G A G T C A G T C G T C A C A G T C T A G T C A G G T A C C T G A T C G A G A T C T G A C | Rfx2(HTH)/LoVo-RFX2-ChIP-Seq(GSE49402)/Homer | 1e-18 | -4.236e+01 | 0.0000 | 745.0 | 5.59% | 796.6 | 4.00% | motif file (matrix) | svg |
| 51 | G C T A G A C T G A C T T G C A C G T A A G T C C G T A T A G C G A T C G A C T | Eomes(T-box)/H9-Eomes-ChIP-Seq(GSE26097)/Homer | 1e-18 | -4.147e+01 | 0.0000 | 7999.0 | 59.99% | 11205.7 | 56.24% | motif file (matrix) | svg |
| 52 | A G T C A T C G C T A G A G C T G A C T C T A G A G T C A G T C G C T A C A G T T C A G T C A G G A T C C T G A T C G A G A T C | RFX(HTH)/K562-RFX3-ChIP-Seq(SRA012198)/Homer | 1e-16 | -3.903e+01 | 0.0000 | 663.0 | 4.97% | 704.0 | 3.53% | motif file (matrix) | svg |
| 53 | A G T C A C G T A C T G A G C T A C G T A C G T G T C A A G T C | Foxo1(Forkhead)/RAW-Foxo1-ChIP-Seq(Fan\_et\_al.)/Homer | 1e-16 | -3.863e+01 | 0.0000 | 7232.0 | 54.24% | 10079.4 | 50.59% | motif file (matrix) | svg |
| 54 | A G T C G A C T C A G T A C T G C T A G T G A C G C T A A T G C G C A T A T C G C G A T A C T G G A T C G T A C G T C A C T G A | NF1(CTF)/LNCAP-NF1-ChIP-Seq(Unpublished)/Homer | 1e-15 | -3.676e+01 | 0.0000 | 1355.0 | 10.16% | 1623.9 | 8.15% | motif file (matrix) | svg |
| 55 | C G A T T A C G T G A C G A C T C A T G C G T A T A C G A C G T G T A C C T G A | Bach2(bZIP)/OCILy7-Bach2-ChIP-Seq(GSE44420)/Homer | 1e-15 | -3.509e+01 | 0.0000 | 1095.0 | 8.21% | 1284.0 | 6.44% | motif file (matrix) | svg |
| 56 | T A G C G T A C A G T C G T A C C G A T A G T C A G T C A G T C A G T C A G T C C G T A G A T C | Zfp281(Zf)/ES-Zfp281-ChIP-Seq(GSE81042)/Homer | 1e-14 | -3.382e+01 | 0.0000 | 623.0 | 4.67% | 672.5 | 3.38% | motif file (matrix) | svg |
| 57 | C A T G C T A G A G C T G A C T C A T G A G T C G A T C G C T A C G A T C T A G T C A G G T A C C T G A T C G A | X-box(HTH)/NPC-H3K4me1-ChIP-Seq(GSE16256)/Homer | 1e-13 | -3.219e+01 | 0.0000 | 848.0 | 6.36% | 970.1 | 4.87% | motif file (matrix) | svg |
| 58 | A G T C C T A G C T A G A G T C G A T C G T A C A G T C C T A G A G T C A G T C A G T C G T A C | Sp2(Zf)/HEK293-Sp2.eGFP-ChIP-Seq(Encode)/Homer | 1e-13 | -3.177e+01 | 0.0000 | 2764.0 | 20.73% | 3617.0 | 18.15% | motif file (matrix) | svg |
| 59 | C T G A C G A T C T A G C G T A A G C T C G A T C A G T C T G A G A C T C T A G C T A G A T G C | PBX2(Homeobox)/K562-PBX2-ChIP-Seq(Encode)/Homer | 1e-13 | -3.143e+01 | 0.0000 | 3922.0 | 29.41% | 5279.5 | 26.50% | motif file (matrix) | svg |
| 60 | A T G C G A C T A C G T C T A G A C G T A C G T A C G T C T G A G A T C G C T A A G C T C G T A | Foxa2(Forkhead)/Liver-Foxa2-ChIP-Seq(GSE25694)/Homer | 1e-12 | -2.986e+01 | 0.0000 | 4001.0 | 30.01% | 5409.6 | 27.15% | motif file (matrix) | svg |
| 61 | C T G A C T A G A C T G G C A T C T A G G C A T A T C G C T G A C G T A G T C A | Tbx21(T-box)/GM12878-TBX21-ChIP-Seq(Encode)/Homer | 1e-12 | -2.938e+01 | 0.0000 | 4379.0 | 32.84% | 5963.0 | 29.93% | motif file (matrix) | svg |
| 62 | C G T A C T A G C A T G A G C T A C T G C G A T A T C G C G T A G T C A G T C A | Tbet(T-box)/CD8-Tbet-ChIP-Seq(GSE33802)/Homer | 1e-10 | -2.449e+01 | 0.0000 | 4567.0 | 34.25% | 6292.6 | 31.58% | motif file (matrix) | svg |
| 63 | T A C G T A G C G C T A C G A T C T A G A C G T C A G T C A G T G C T A A G T C G T C A G C A T | FOXK2(Forkhead)/U2OS-FOXK2-ChIP-Seq(E-MTAB-2204)/Homer | 1e-10 | -2.432e+01 | 0.0000 | 3165.0 | 23.74% | 4259.9 | 21.38% | motif file (matrix) | svg |
| 64 | A C G T C T A G A G C T A C G T A C G T C T G A A G T C G A C T A G C T C G T A | FOXM1(Forkhead)/MCF7-FOXM1-ChIP-Seq(GSE72977)/Homer | 1e-10 | -2.400e+01 | 0.0000 | 5109.0 | 38.32% | 7093.5 | 35.60% | motif file (matrix) | svg |
| 65 | C T A G T A C G G A T C G T A C G C T A A G C T A G C T G T C A T C G A T A G C | Nanog(Homeobox)/mES-Nanog-ChIP-Seq(GSE11724)/Homer | 1e-9 | -2.252e+01 | 0.0000 | 10970.0 | 82.27% | 15965.5 | 80.13% | motif file (matrix) | svg |
| 66 | T C G A T G C A A C T G G T C A A G T C A G T C A G T C A G T C A G C T T G A C | LRF(Zf)/Erythroblasts-ZBTB7A-ChIP-Seq(GSE74977)/Homer | 1e-9 | -2.163e+01 | 0.0000 | 2403.0 | 18.02% | 3195.7 | 16.04% | motif file (matrix) | svg |
| 67 | A G T C T A G C G A C T A C G T C T A G A C G T A C G T A C G T C T G A A G T C G C T A G A C T C G T A C T A G A C T G | Foxa3(Forkhead)/Liver-Foxa3-ChIP-Seq(GSE77670)/Homer | 1e-9 | -2.148e+01 | 0.0000 | 1839.0 | 13.79% | 2397.4 | 12.03% | motif file (matrix) | svg |
| 68 | G T A C G C A T C T G A C G T A G A C T A G C T C A T G T G C A C T G A A C G T G A C T C G T A | Prop1(Homeobox)/GHFT1-PROP1.biotin-ChIP-Seq(GSE77302)/Homer | 1e-9 | -2.137e+01 | 0.0000 | 3564.0 | 26.73% | 4868.9 | 24.44% | motif file (matrix) | svg |
| 69 | C G A T C T A G T C G A A G C T C G A T C T G A C G T A A G C T A C T G C T A G A T G C G A T C | Hoxb4(Homeobox)/ES-Hoxb4-ChIP-Seq(GSE34014)/Homer | 1e-8 | -2.044e+01 | 0.0000 | 903.0 | 6.77% | 1106.8 | 5.55% | motif file (matrix) | svg |
| 70 | C T A G A C G T G C A T T C G A G T C A G C A T A T C G C G T A C A G T A G C T C T G A T G C A | HNF1b(Homeobox)/PDAC-HNF1B-ChIP-Seq(GSE64557)/Homer | 1e-8 | -1.971e+01 | 0.0000 | 792.0 | 5.94% | 960.1 | 4.82% | motif file (matrix) | svg |
| 71 | T C A G T A C G T A G C A C G T A C T G C G A T A G T C C G T A T A C G A G T C | Meis1(Homeobox)/MastCells-Meis1-ChIP-Seq(GSE48085)/Homer | 1e-7 | -1.828e+01 | 0.0000 | 5643.0 | 42.32% | 7959.0 | 39.94% | motif file (matrix) | svg |
| 72 | T G C A C T G A A T G C G T C A A C G T A T G C A C G T A C T G A C T G T G C A | ZBTB18(Zf)/HEK293-ZBTB18.GFP-ChIP-Seq(GSE58341)/Homer | 1e-7 | -1.726e+01 | 0.0000 | 2171.0 | 16.28% | 2909.5 | 14.60% | motif file (matrix) | svg |
| 73 | C G A T C T G A C G T A A G C T A G T C T G C A C T G A A C G T A G C T G C T A | Pax7(Paired,Homeobox)/Myoblast-Pax7-ChIP-Seq(GSE25064)/Homer | 1e-7 | -1.690e+01 | 0.0000 | 547.0 | 4.10% | 647.9 | 3.25% | motif file (matrix) | svg |
| 74 | C T A G C A G T C G T A A C G T A G T C A C T G C G T A A G C T A G T C G A T C | HNF6(Homeobox)/Liver-Hnf6-ChIP-Seq(ERP000394)/Homer | 1e-7 | -1.678e+01 | 0.0000 | 1969.0 | 14.77% | 2626.9 | 13.18% | motif file (matrix) | svg |
| 75 | G C T A A C T G T C G A C T G A C T G A A C G T T A G C C T G A C G T A C G A T | Cux2(Homeobox)/Liver-Cux2-ChIP-Seq(GSE35985)/Homer | 1e-7 | -1.670e+01 | 0.0000 | 1652.0 | 12.39% | 2178.0 | 10.93% | motif file (matrix) | svg |
| 76 | T C G A C T G A C T G A A T G C G T A C G T C A A G C T A G C T C G T A T C A G | Barx1(Homeobox)/Stomach-Barx1.3xFlag-ChIP-Seq(GSE69483)/Homer | 1e-7 | -1.646e+01 | 0.0000 | 2606.0 | 19.54% | 3542.2 | 17.78% | motif file (matrix) | svg |
| 77 | A C T G A C G T C A T G A T C G A T C G T G A C A C T G A T C G A T C G T G C A C T G A C G T A | E2F3(E2F)/MEF-E2F3-ChIP-Seq(GSE71376)/Homer | 1e-6 | -1.513e+01 | 0.0000 | 957.0 | 7.18% | 1217.2 | 6.11% | motif file (matrix) | svg |
| 78 | C T A G C T G A T C A G A T C G A G C T C A T G G A C T A G T C C T G A T G C A | Tbx6(T-box)/ESC-Tbx6-ChIP-Seq(GSE93524)/Homer | 1e-6 | -1.496e+01 | 0.0000 | 4230.0 | 31.72% | 5925.4 | 29.74% | motif file (matrix) | svg |
| 79 | T A C G C T A G A T G C G A T C G T A C A G T C C T A G A G T C A G T C A G T C G T A C A G T C | Sp1(Zf)/Promoter/Homer | 1e-6 | -1.492e+01 | 0.0000 | 241.0 | 1.81% | 257.9 | 1.29% | motif file (matrix) | svg |
| 80 | T A C G A T G C G A C T A C T G A G C T A G T C G T C A T G C A A C G T A G T C G C T A T G C A | Pknox1(Homeobox)/ES-Prep1-ChIP-Seq(GSE63282)/Homer | 1e-6 | -1.491e+01 | 0.0000 | 861.0 | 6.46% | 1086.6 | 5.45% | motif file (matrix) | svg |
| 81 | A T G C G T A C C G T A A G C T G C A T T A C G A G C T A G C T A G T C A G C T | Sox6(HMG)/Myotubes-Sox6-ChIP-Seq(GSE32627)/Homer | 1e-6 | -1.462e+01 | 0.0000 | 6124.0 | 45.93% | 8729.5 | 43.81% | motif file (matrix) | svg |
| 82 | G C A T C G T A C G A T G A C T A C T G C T G A G A C T G A T C | Hnf6b(Homeobox)/LNCaP-Hnf6b-ChIP-Seq(GSE106305)/Homer | 1e-6 | -1.461e+01 | 0.0000 | 3324.0 | 24.93% | 4606.9 | 23.12% | motif file (matrix) | svg |
| 83 | C T A G T A C G G A T C G T A C C T G A A G C T T G C A G C T A C G T A G C A T G A T C G C T A | Hoxc9(Homeobox)/Ainv15-Hoxc9-ChIP-Seq(GSE21812)/Homer | 1e-6 | -1.420e+01 | 0.0000 | 2531.0 | 18.98% | 3462.7 | 17.38% | motif file (matrix) | svg |
| 84 | C G A T A C G T A C G T A C G T C G T A A G C T C A G T C T A G A T C G A C T G | HOXB13(Homeobox)/ProstateTumor-HOXB13-ChIP-Seq(GSE56288)/Homer | 1e-6 | -1.415e+01 | 0.0000 | 4943.0 | 37.07% | 6987.4 | 35.07% | motif file (matrix) | svg |
| 85 | T A G C T A G C G A C T C T A G A G C T A G T C G T C A T G C A A C G T A T G C G C T A T G C A | Pbx3(Homeobox)/GM12878-PBX3-ChIP-Seq(GSE32465)/Homer | 1e-6 | -1.408e+01 | 0.0000 | 951.0 | 7.13% | 1217.5 | 6.11% | motif file (matrix) | svg |
| 86 | C G T A C G T A C G T A G C A T G C A T T A C G G T A C G A C T A C T G C G T A A T C G A C G T G T A C C G T A A G C T | Bach1(bZIP)/K562-Bach1-ChIP-Seq(GSE31477)/Homer | 1e-6 | -1.392e+01 | 0.0000 | 371.0 | 2.78% | 429.7 | 2.16% | motif file (matrix) | svg |
| 87 | C G A T C T A G A C G T A C G T A C G T C G T A A G C T C G A T A G C T C G T A C T A G T A G C | FoxD3(forkhead)/ZebrafishEmbryo-Foxd3.biotin-ChIP-seq(GSE106676)/Homer | 1e-5 | -1.380e+01 | 0.0000 | 4443.0 | 33.32% | 6256.9 | 31.40% | motif file (matrix) | svg |
| 88 | T A G C C G A T A C G T A G C T A G C T A G T C A T G C A G T C A C T G A T G C A T G C G C T A | E2F7(E2F)/Hela-E2F7-ChIP-Seq(GSE32673)/Homer | 1e-5 | -1.365e+01 | 0.0000 | 111.0 | 0.83% | 102.1 | 0.51% | motif file (matrix) | svg |
| 89 | G A C T C T A G C T A G C T A G A C T G T C G A C T G A C T A G C T A G C T A G G T A C G T C A | ZNF467(Zf)/HEK293-ZNF467.GFP-ChIP-Seq(GSE58341)/Homer | 1e-5 | -1.329e+01 | 0.0000 | 2146.0 | 16.09% | 2919.4 | 14.65% | motif file (matrix) | svg |
| 90 | A T G C A G T C G A T C C G T A A C G T A C G T A C T G A C G T A G C T G A T C | Sox2(HMG)/mES-Sox2-ChIP-Seq(GSE11431)/Homer | 1e-5 | -1.294e+01 | 0.0000 | 3408.0 | 25.56% | 4753.9 | 23.86% | motif file (matrix) | svg |
| 91 | A T G C G A T C C G T A A G C T C A G T A T C G G C A T A G C T G A C T A C T G | Sox17(HMG)/Endoderm-Sox17-ChIP-Seq(GSE61475)/Homer | 1e-5 | -1.270e+01 | 0.0000 | 2874.0 | 21.55% | 3979.9 | 19.97% | motif file (matrix) | svg |
| 92 | T G A C T A G C T C A G T C G A T C G A C G T A A G T C C G T A C G T A C G A T C T A G T A C G | Sox7(HMG)/ESC-Sox7-ChIP-Seq(GSE133899)/Homer | 1e-5 | -1.246e+01 | 0.0000 | 1151.0 | 8.63% | 1511.9 | 7.59% | motif file (matrix) | svg |
| 93 | C T G A C T G A C T A G T C G A C G T A A T G C C G T A A C T G C G T A A C G T C T G A C G A T A G C T C G T A A C G T A G T C C G A T T A C G G T C A G C A T | GATA(Zf),IR3/iTreg-Gata3-ChIP-Seq(GSE20898)/Homer | 1e-5 | -1.217e+01 | 0.0000 | 712.0 | 5.34% | 901.4 | 4.52% | motif file (matrix) | svg |
| 94 | C T A G G T A C A G T C T G C A A G T C C T G A A G T C A G T C A G T C G C T A | Klf4(Zf)/mES-Klf4-ChIP-Seq(GSE11431)/Homer | 1e-5 | -1.172e+01 | 0.0000 | 845.0 | 6.34% | 1089.6 | 5.47% | motif file (matrix) | svg |
| 95 | T A C G T C A G T G C A A G C T T G A C A G C T A G T C A C T G G A T C A C T G T C G A A C T G C T G A C T G A A T G C | ZBTB33(Zf)/GM12878-ZBTB33-ChIP-Seq(GSE32465)/Homer | 1e-5 | -1.166e+01 | 0.0000 | 29.0 | 0.22% | 17.6 | 0.09% | motif file (matrix) | svg |
| 96 | G A C T G C A T C T A G C G A T G A T C T C G A C A T G G A T C | Tgif1(Homeobox)/mES-Tgif1-ChIP-Seq(GSE55404)/Homer | 1e-5 | -1.162e+01 | 0.0000 | 9432.0 | 70.74% | 13753.0 | 69.02% | motif file (matrix) | svg |
| 97 | G T A C C A G T A C T G A C T G A C T G G A T C A C T G A C G T A C T G A C T G A G T C G A T C | KLF6(Zf)/PDAC-KLF6-ChIP-Seq(GSE64557)/Homer | 1e-4 | -1.142e+01 | 0.0000 | 1991.0 | 14.93% | 2720.4 | 13.65% | motif file (matrix) | svg |
| 98 | T A C G T C A G G A T C G T A C T C G A G A C T G C T A G C T A G C T A C G T A G A T C G T C A | CDX4(Homeobox)/ZebrafishEmbryos-Cdx4.Myc-ChIP-Seq(GSE48254)/Homer | 1e-4 | -1.138e+01 | 0.0001 | 4335.0 | 32.51% | 6138.4 | 30.81% | motif file (matrix) | svg |
| 99 | A T G C C G T A A C T G C G T A A C G T G C T A T C G A A G C T C G A T C G T A A C G T A G T C C G A T A C T G G A T C | GATA(Zf),IR4/iTreg-Gata3-ChIP-Seq(GSE20898)/Homer | 1e-4 | -1.102e+01 | 0.0001 | 359.0 | 2.69% | 428.0 | 2.15% | motif file (matrix) | svg |
| 100 | G A C T T C A G C T A G A G T C A G T C G T A C A G T C C T G A A G T C A G T C A G T C G A C T A G T C A C T G A T G C | KLF3(Zf)/MEF-Klf3-ChIP-Seq(GSE44748)/Homer | 1e-4 | -1.100e+01 | 0.0001 | 902.0 | 6.76% | 1175.9 | 5.90% | motif file (matrix) | svg |
| 101 | C T A G A T G C A T G C C G A T A C T G G A C T A T G C G C T A T G A C A G C T T A G C G C T A | PBX1(Homeobox)/MCF7-PBX1-ChIP-Seq(GSE28007)/Homer | 1e-4 | -1.085e+01 | 0.0001 | 317.0 | 2.38% | 373.2 | 1.87% | motif file (matrix) | svg |
| 102 | C T A G T A C G G A T C G T C A T G C A A C G T T G C A G C T A T C G A T G C A | Hoxa9(Homeobox)/ChickenMSG-Hoxa9.Flag-ChIP-Seq(GSE86088)/Homer | 1e-4 | -1.083e+01 | 0.0001 | 9125.0 | 68.43% | 13302.1 | 66.76% | motif file (matrix) | svg |
| 103 | T A C G C T G A C T A G T C G A C G T A A G T C C T G A A T C G G C A T T A G C G A C T A C T G A C G T A G C T A G T C G A C T | GRE(NR),IR3/A549-GR-ChIP-Seq(GSE32465)/Homer | 1e-4 | -1.053e+01 | 0.0001 | 528.0 | 3.96% | 660.8 | 3.32% | motif file (matrix) | svg |
| 104 | T C A G A G C T G T A C C G T A A C G T C G T A C G T A C G T A G C T A G A C T | Cdx2(Homeobox)/mES-Cdx2-ChIP-Seq(GSE14586)/Homer | 1e-4 | -1.050e+01 | 0.0001 | 3532.0 | 26.49% | 4974.7 | 24.97% | motif file (matrix) | svg |
| 105 | G T C A G C A T A C T G G T A C G A C T A C T G G C T A A T C G C A G T G T A C C G T A A G C T | Nrf2(bZIP)/Lymphoblast-Nrf2-ChIP-Seq(GSE37589)/Homer | 1e-4 | -1.031e+01 | 0.0001 | 287.0 | 2.15% | 336.3 | 1.69% | motif file (matrix) | svg |
| 106 | T A G C C G A T T A C G A C T G A G T C A C T G A T C G A T C G C G T A C T G A | E2F1(E2F)/Hela-E2F1-ChIP-Seq(GSE22478)/Homer | 1e-4 | -1.028e+01 | 0.0001 | 202.0 | 1.51% | 225.9 | 1.13% | motif file (matrix) | svg |
| 107 | A G T C A T C G G C A T C T A G A C T G T A C G C G A T T C A G C A T G A G C T T A G C G A T C | GLI3(Zf)/Limb-GLI3-ChIP-Chip(GSE11077)/Homer | 1e-4 | -1.023e+01 | 0.0001 | 298.0 | 2.23% | 351.6 | 1.76% | motif file (matrix) | svg |
| 108 | C G T A C T A G A C T G A C T G G A C T C T A G C A G T C T A G C A T G G A T C | KLF5(Zf)/LoVo-KLF5-ChIP-Seq(GSE49402)/Homer | 1e-4 | -1.017e+01 | 0.0002 | 2516.0 | 18.87% | 3497.2 | 17.55% | motif file (matrix) | svg |
| 109 | C T A G T A G C A G T C G T C A C T G A A C G T C G T A C G T A C G T A G C T A | Hoxd13(Homeobox)/ChickenMSG-Hoxd13.Flag-ChIP-Seq(GSE86088)/Homer | 1e-4 | -1.003e+01 | 0.0002 | 7201.0 | 54.00% | 10422.5 | 52.31% | motif file (matrix) | svg |
| 110 | C G T A C T G A C T A G C G T A C G T A A G T C C G T A C A G T G C A T G T C A C G A T A C T G A C G T G C A T G A T C | PGR(NR)/EndoStromal-PGR-ChIP-Seq(GSE69539)/Homer | 1e-4 | -9.852e+00 | 0.0002 | 1275.0 | 9.56% | 1714.2 | 8.60% | motif file (matrix) | svg |
| 111 | T G A C C G A T A C T G A C T G A C T G G A C T A C T G A C G T A C T G A C T G G A T C G A T C | EKLF(Zf)/Erythrocyte-Klf1-ChIP-Seq(GSE20478)/Homer | 1e-4 | -9.616e+00 | 0.0003 | 537.0 | 4.03% | 679.7 | 3.41% | motif file (matrix) | svg |
| 112 | G A T C G C A T C T G A C G T A A G C T A G C T C G T A T C G A C T G A A C G T G C A T C G T A | Phox2a(Homeobox)/Neuron-Phox2a-ChIP-Seq(GSE31456)/Homer | 1e-4 | -9.544e+00 | 0.0003 | 2264.0 | 16.98% | 3141.6 | 15.77% | motif file (matrix) | svg |
| 113 | T C A G C T A G C T A G A C T G A C T G G A T C A C T G A C T G C T A G C T A G A G T C G A T C | KLF1(Zf)/HUDEP2-KLF1-CutnRun(GSE136251)/Homer | 1e-3 | -9.124e+00 | 0.0004 | 1572.0 | 11.79% | 2148.8 | 10.78% | motif file (matrix) | svg |
| 114 | C G T A C G T A C G T A G C A T G C A T A C T G G T A C G A C T C T A G G C T A T A C G G A C T T G A C C G T A A G C T | NFE2L2(bZIP)/HepG2-NFE2L2-ChIP-Seq(Encode)/Homer | 1e-3 | -9.021e+00 | 0.0005 | 396.0 | 2.97% | 490.0 | 2.46% | motif file (matrix) | svg |
| 115 | T A C G T C G A C A G T A C T G G C T A A T G C C G A T G T A C C G T A A C T G T A G C C G T A | NF-E2(bZIP)/K562-NFE2-ChIP-Seq(GSE31477)/Homer | 1e-3 | -8.724e+00 | 0.0006 | 346.0 | 2.59% | 424.8 | 2.13% | motif file (matrix) | svg |
| 116 | A G T C A T C G G C A T C A T G A C T G A T C G C G A T C T A G A C T G A G C T T G A C G A C T | Gli2(Zf)/GM2-Gli2-ChIP-Chip(GSE112702)/Homer | 1e-3 | -8.709e+00 | 0.0006 | 642.0 | 4.81% | 832.2 | 4.18% | motif file (matrix) | svg |
| 117 | T C G A T A G C T G A C C T G A A G T C A C T G G A C T C A T G | c-Myc(bHLH)/LNCAP-cMyc-ChIP-Seq(Unpublished)/Homer | 1e-3 | -8.672e+00 | 0.0006 | 1006.0 | 7.54% | 1345.9 | 6.75% | motif file (matrix) | svg |
| 118 | C G A T A C G T C G T A C G T A A C G T A G C T T G C A C T G A C G T A A C G T A C G T C G T A | Phox2b(Homeobox)/CLBGA-PHOX2B-ChIP-Seq(GSE90683)/Homer | 1e-3 | -8.041e+00 | 0.0012 | 1242.0 | 9.31% | 1689.8 | 8.48% | motif file (matrix) | svg |
| 119 | A G T C C G A T A C T G A T C G T G A C G C T A C A T G A T C G T G A C C G A T A C T G T A G C G T A C G T C A | Tlx?(NR)/NPC-H3K4me1-ChIP-Seq(GSE16256)/Homer | 1e-3 | -7.866e+00 | 0.0014 | 1416.0 | 10.62% | 1941.6 | 9.74% | motif file (matrix) | svg |
| 120 | T G A C C T A G A C T G T A G C C G A T A C T G A T G C C A T G A T C G A T C G A T C G T A G C C T G A T A G C G C T A A C T G C G T A A G C T C G T A C T G A | GATA:SCL(Zf,bHLH)/Ter119-SCL-ChIP-Seq(GSE18720)/Homer | 1e-3 | -7.726e+00 | 0.0016 | 493.0 | 3.70% | 633.3 | 3.18% | motif file (matrix) | svg |
| 121 | T C G A C A T G C A T G T A C G G T A C T C G A A G T C C A G T C T G A C G T A A G T C G A C T | ZNF264(Zf)/HEK293-ZNF264.GFP-ChIP-Seq(GSE58341)/Homer | 1e-3 | -7.487e+00 | 0.0020 | 1838.0 | 13.78% | 2556.3 | 12.83% | motif file (matrix) | svg |
| 122 | A T G C A G T C G C A T A G C T A C G T T C A G C G A T A G C T G A T C A T C G | Sox10(HMG)/SciaticNerve-Sox3-ChIP-Seq(GSE35132)/Homer | 1e-3 | -7.377e+00 | 0.0023 | 5792.0 | 43.44% | 8379.5 | 42.05% | motif file (matrix) | svg |
| 123 | T A C G T A C G G T A C A T C G T A C G T A C G G T C A C T G A C G T A G A C T | E2F4(E2F)/K562-E2F4-ChIP-Seq(GSE31477)/Homer | 1e-3 | -7.167e+00 | 0.0028 | 481.0 | 3.61% | 621.5 | 3.12% | motif file (matrix) | svg |
| 124 | G A T C G C T A C A G T A C G T T A C G A G T C A T G C C T A G A G T C T C G A | Zfp57(Zf)/H1-ZFP57.HA-ChIP-Seq(GSE115387)/Homer | 1e-3 | -7.142e+00 | 0.0028 | 483.0 | 3.62% | 624.9 | 3.14% | motif file (matrix) | svg |
| 125 | A T G C A T G C A T C G T A C G A G C T A G T C G C T A A G T C T C A G G A C T A C T G T C G A | E-box(bHLH)/Promoter/Homer | 1e-2 | -6.673e+00 | 0.0045 | 156.0 | 1.17% | 181.1 | 0.91% | motif file (matrix) | svg |
| 126 | A T G C A T C G T A C G A G C T A T C G C T G A A G T C C T A G A G C T A T G C C T G A A T G C | CRE(bZIP)/Promoter/Homer | 1e-2 | -6.523e+00 | 0.0051 | 559.0 | 4.19% | 736.5 | 3.70% | motif file (matrix) | svg |
| 127 | C T G A T C G A C G T A A T G C C G T A C G T A C G A T C T A G T C A G G A T C | Sox15(HMG)/CPA-Sox15-ChIP-Seq(GSE62909)/Homer | 1e-2 | -6.465e+00 | 0.0054 | 4137.0 | 31.03% | 5947.5 | 29.85% | motif file (matrix) | svg |
| 128 | C G T A C G A T G A C T G A C T T G A C C T G A A T G C C T G A T A G C A G T C A C G T T C G A C A T G T A C G G A C T A T C G G A C T A C G T C T G A T C G A C G T A | Brachyury(T-box)/Mesoendoderm-Brachyury-ChIP-exo(GSE54963)/Homer | 1e-2 | -6.364e+00 | 0.0059 | 1305.0 | 9.79% | 1803.2 | 9.05% | motif file (matrix) | svg |
| 129 | G A C T C T A G G A T C C A G T A C T G C T G A A T G C G C A T A T G C C T G A | MafA(bZIP)/Islet-MafA-ChIP-Seq(GSE30298)/Homer | 1e-2 | -6.300e+00 | 0.0063 | 2884.0 | 21.63% | 4105.2 | 20.60% | motif file (matrix) | svg |
| 130 | C G T A C T A G T C A G T C A G A G T C A T G C A G T C G C A T A G C T A C G T A T C G C G A T | Sox9(HMG)/Limb-SOX9-ChIP-Seq(GSE73225)/Homer | 1e-2 | -6.248e+00 | 0.0065 | 3199.0 | 23.99% | 4569.5 | 22.93% | motif file (matrix) | svg |
| 131 | T C G A G C A T A T G C C T G A A T G C T A G C A G T C G T A C T C G A A G C T | Srebp1a(bHLH)/HepG2-Srebp1a-ChIP-Seq(GSE31477)/Homer | 1e-2 | -6.192e+00 | 0.0069 | 607.0 | 4.55% | 807.6 | 4.05% | motif file (matrix) | svg |
| 132 | C A T G G T A C C G T A A G T C C T A G A C G T A C T G G T A C A G T C A G C T | bHLHE40(bHLH)/HepG2-BHLHE40-ChIP-Seq(GSE31477)/Homer | 1e-2 | -6.101e+00 | 0.0075 | 741.0 | 5.56% | 999.0 | 5.01% | motif file (matrix) | svg |
| 133 | T C A G C A T G C A T G A C T G A C T G A G C T A C T G A C G T A C T G C A G T A T G C A G T C | KLF10(Zf)/HEK293-KLF10.GFP-ChIP-Seq(GSE58341)/Homer | 1e-2 | -6.071e+00 | 0.0076 | 2008.0 | 15.06% | 2828.6 | 14.20% | motif file (matrix) | svg |
| 134 | T G C A A G C T C T G A A T C G G A C T C T A G G T A C G A T C G T C A A G T C G T A C G A C T C T A G A T C G G C A T C A T G C A T G G A T C G T A C C T G A | CTCF(Zf)/CD4+-CTCF-ChIP-Seq(Barski\_et\_al.)/Homer | 1e-2 | -6.005e+00 | 0.0081 | 277.0 | 2.08% | 348.5 | 1.75% | motif file (matrix) | svg |
| 135 | C T A G G A C T G A T C A C G T A T C G A G C T C T G A A T C G C G A T C T A G G A T C G A C T C A T G A T C G G T A C G A C T A G T C G C A T A G C T C G A T | ZNF382(Zf)/HEK293-ZNF382.GFP-ChIP-Seq(GSE58341)/Homer | 1e-2 | -5.958e+00 | 0.0084 | 103.0 | 0.77% | 115.5 | 0.58% | motif file (matrix) | svg |
| 136 | A T G C G A T C C G A T A C G T A C G T A C T G C A G T A G C T | Sox3(HMG)/NPC-Sox3-ChIP-Seq(GSE33059)/Homer | 1e-2 | -5.916e+00 | 0.0087 | 6174.0 | 46.30% | 8986.7 | 45.10% | motif file (matrix) | svg |
| 137 | G A C T G A C T T C G A C G T A C G A T A G C T C T G A A C T G T G A C G A C T T C G A C G T A A C G T A G C T C T G A C T G A G T C A G C T A G C T A C G T A | Pax7(Paired,Homeobox),longest/Myoblast-Pax7-ChIP-Seq(GSE25064)/Homer | 1e-2 | -5.755e+00 | 0.0102 | 198.0 | 1.48% | 242.1 | 1.21% | motif file (matrix) | svg |
| 138 | T G A C C T G A C T A G C T G A C G T A A G T C C T G A A C G T G C A T T A G C G C A T A T C G G A C T G A C T G A T C | GRE(NR),IR3/RAW264.7-GRE-ChIP-Seq(Unpublished)/Homer | 1e-2 | -5.494e+00 | 0.0131 | 1035.0 | 7.76% | 1427.2 | 7.16% | motif file (matrix) | svg |
| 139 | T C A G T A G C G A C T C A T G C T G A A T C G G C A T G T A C C G T A A C T G T A G C T G C A | MafK(bZIP)/C2C12-MafK-ChIP-Seq(GSE36030)/Homer | 1e-2 | -5.338e+00 | 0.0152 | 918.0 | 6.88% | 1261.2 | 6.33% | motif file (matrix) | svg |
| 140 | G C A T T C G A C T G A G A T C A G T C G A T C G T C A G T C A A C G T A G T C C G T A C T G A | Duxbl(Homeobox)/NIH3T3-Duxbl.HA-ChIP-Seq(GSE119782)/Homer | 1e-2 | -5.275e+00 | 0.0161 | 320.0 | 2.40% | 413.5 | 2.08% | motif file (matrix) | svg |
| 141 | A G T C G C A T C G T A C G T A G T A C A C G T A C T G G A T C G A T C T C G A | BMYB(HTH)/Hela-BMYB-ChIP-Seq(GSE27030)/Homer | 1e-2 | -5.067e+00 | 0.0197 | 5425.0 | 40.69% | 7895.6 | 39.63% | motif file (matrix) | svg |
| 142 | T A C G T A C G G T A C A T C G A C T G T A C G T C G A C T G A T C G A A T C G | E2F6(E2F)/Hela-E2F6-ChIP-Seq(GSE31477)/Homer | 1e-2 | -5.038e+00 | 0.0201 | 738.0 | 5.53% | 1007.1 | 5.05% | motif file (matrix) | svg |
| 143 | A T G C T C A G T C G A G C A T A C T G C G T A A G T C T C A G G A C T T G A C C G T A A G C T | Atf2(bZIP)/3T3L1-Atf2-ChIP-Seq(GSE56872)/Homer | 1e-2 | -4.872e+00 | 0.0236 | 1101.0 | 8.26% | 1532.4 | 7.69% | motif file (matrix) | svg |
| 144 | T C A G C G T A A G T C A G C T C G T A A G T C C T G A C G T A A G T C G C A T A G T C A G T C A G T C C T G A A C T G T G C A T C G A C A T G A T C G G A T C | Ronin(THAP)/ES-Thap11-ChIP-Seq(GSE51522)/Homer | 1e-2 | -4.851e+00 | 0.0239 | 33.0 | 0.25% | 31.9 | 0.16% | motif file (matrix) | svg |
| 145 | A T G C C T G A A T C G T A C G A G T C C G A T T C A G C G A T C T A G A G C T G T C A G T C A C G T A A G T C C G T A T A C G C T G A | Fox:Ebox(Forkhead,bHLH)/Panc1-Foxa2-ChIP-Seq(GSE47459)/Homer | 1e-2 | -4.749e+00 | 0.0263 | 4439.0 | 33.29% | 6440.0 | 32.32% | motif file (matrix) | svg |
| 146 | C T G A C T A G T C G A C G T A A T G C C G T A A T C G C G A T T A G C G C A T A T C G G C A T A G C T G A T C G A C T A G C T | ARE(NR)/LNCAP-AR-ChIP-Seq(GSE27824)/Homer | 1e-2 | -4.695e+00 | 0.0275 | 980.0 | 7.35% | 1360.6 | 6.83% | motif file (matrix) | svg |
| 147 | C T A G G A T C T C G A A T G C T C G A A G C T C T G A A C T G A C G T C T G A A C T G A C T G A C G T A G T C G A T C | ZKSCAN1(Zf)/HepG2-ZKSCAN1-ChIP-Seq(Encode)/Homer | 1e-2 | -4.663e+00 | 0.0283 | 85.0 | 0.64% | 97.4 | 0.49% | motif file (matrix) | svg |
| 148 | T C A G A T C G G A C T A C T G G A C T C A G T C T A G C G T A G T A C C G T A C T A G A T C G | Tbx20(T-box)/Heart-Tbx20-ChIP-Seq(GSE29636)/Homer | 1e-2 | -4.639e+00 | 0.0287 | 824.0 | 6.18% | 1136.1 | 5.70% | motif file (matrix) | svg |
